# Gout and Goutiness

**Published:** 1897-06

**Authors:** 


**Gout and Goutiness.** By William Ewart, M.D. Pp. xii., 589. London: Baillière, Tindall and Cox. 1896.

Dr. Ewart's book embraces all theories of gout. Every aspect of the disease has been worked out by him with an accuracy that is beyond praise. That the uric acid diathesis embraces a great deal more than gout, that it is a question whether gout has any separate existence apart from changes in the joints, that goutiness, with all its nervous phenomena, more common in women than the acute disease, exists as an independent entity, that there is an attenuation of types, and a pre-arthritic stage in gout, all these points force the conclusion that we can hardly deny the notion of a general and constitutional affection.

Gout is a perversion of nutrition. Buzzard believes that uratic deposits may occur in the lymphatics of a peripheral nerve, and that a local neuritis may be the result. Wade's neural theory of gout is founded upon this assumption; but although there is evidence of the priority of the nerve disorder in the sensory, vaso-motor, and trophic changes, the missing link in the demonstration is the proof of a pre-existing neuritis culminating in an arthritic attack. Formerly the humoral theory held sway; and of late the primary humoral aspect of faulty nutrition has been worked in by Sir Dyce Duckworth, with the secondary dystrophic aspect based upon disordered innervation. Garrod demonstrated the abnormal presence of uric acid in the blood. To him the deposition of the precipitate of the solid sodium biurate crystal was the harmful fact. Dr. Haig's toxic uric acid theory of gout recognises the deleterious action of uric acid when circulating in a soluble state: whilst Ebstein conceives a double and reciprocal influence—toxic on the one hand, mechanical on the other—between the tissues and the biurate. Sir William Roberts's experiments are described at some length; the extent of the solubility of the biurate and quadriurate of sodium, the beneficent influence of common salt, an insufficient supply of which may explain the frequency of uric acid concretions among the Hindoos, whose staple diet is rice, &c. The accumulation of uric acid in the blood depends largely on renal inadequacy. Its normal output is much exceeded in certain diseases, such as leucocythæmia, owing to the vast increase of leucocytes; whilst in simple anæmia, in which the leucocytes are not increased, the amount of uric acid is not abnormal. Sir W. Roberts has suggested that the excretion of uric acid, instead of urea, constitutes a recurrence towards an earlier type of existence; evolution having gradually replaced uric acid by urea.

Dr. Ewart speaks of the connection of gout and rheumatism and rheumatoid arthritis, but without touching on the presence of micro-organisms in the latter ailment. Patients with rheumatoid arthritis are liable to gouty complications. The gouty and the scrofulous constitution may co-exist. Glycosuria usually coincides with a remission of the gouty manifestations. The connection of plumbism and gout is probably due to the influence of lead on the liver and the intestines, rather than in a more direct relation.

The clinical types of the disease are examined with care: the prearthritic stage, the arthritic, the joints affected, chronic gout, and gouty cachexia; and also the clinical features of gout and goutiness in relation to the various organs. The author considers that clinically gouty gastritis is metastatic, but agrees with most observers that probably many of the fatal cases depend on angina pectoris. The urogenital mucous membrane, the organs of reproduction, the liver, kidneys, heart and veins, the skin and the brain may all suffer from gout; but in the brain the affection is metastatic, as is also gouty congestive paraplegia.

Gout may be looked upon too exclusively as a renal, a hepatic, or a nervous question. These three aspects are in reality inseparable. The nervous system has not only an undoubted influence on the metabolic changes and assimilations, but also exercises a determining influence upon some of the acute phenomena of gout.

The author discusses treatment with great fulness. His suggestions may almost be compressed into the dictum, "Live an active and frugal life," using the term in the wide sense of working within one's strength, and being frugal not only in food and drink, but as to all excess in matters connected either with body or mind. Various drugs are passed in review, and the advantages and disadvantges of colchicum touched upon. Salicylates are useful in a minor degree. The nature of the attack and the condition of the patient must influence all treatment. Alkalies do not dissolve the biurate, but perhaps the kidneys are thus assisted in separating uric acid from the blood. Even lithia salts are poor solvents. The treatment of chronic gout is largely one of environment, but guaiacum is useful. Uric acid is formed not only from animal albumen, but from vegetable albumen also, though in a less degree. "Live by the baker, the dairyman, and the fruiterer" (Haig), with a minimum of meat, and with regular and moderate exercise. In gouty glycosuria and diabetes, strict diabetic diet is not appropriate. Those wines agree best which act upon the patient as diuretics. British and foreign spas are passed in review; and the climate of Clifton and the waters of Bath are commended.

The book is an exhaustive study of gout in all its forms, and is characterised throughout by thorough common sense.

